# Disentangling the association between cognitive flexibility and anxiety in autistic youth: real-world flexibility versus performance-based task switching

**DOI:** 10.3389/fpsyt.2025.1570185

**Published:** 2025-07-09

**Authors:** Farah Mahmud, Erin Kang, Rachel G. McDonald, Drew Wallace, Carrie Masia Warner

**Affiliations:** ^1^ Department of Psychiatry, Columbia University Medical Center, New York, NY, United States; ^2^ Division of Child and Adolescent Psychiatry, New York State Psychiatric Institute, New York, NY, United States; ^3^ Psychology Department, Montclair State University, Montclair, NJ, United States; ^4^ University Libraries, Montclair State University, Montclair, NJ, United States; ^5^ Social Solutions and Services Research, Center for Cultural and Structural Equity, Nathan Kline Institute for Psychiatric Research, Orangeburg, NY, United States

**Keywords:** cognitive flexibility, executive function, task switching, anxiety, autism, autistic self-report, children and adolescents, neuropsychological assessment

## Abstract

**Introduction:**

Anxiety symptoms are highly prevalent among autistic youth yet remain under-recognized and undertreated, in part, due to a limited conceptual understanding of autistic cognition. Emerging evidence suggests that autistic differences in cognitive flexibility (CF) may be associated with a greater likelihood of developing and maintaining anxiety symptoms, relative to non-autistic youth. However, further work is needed to elucidate mechanisms of anxiety vulnerability that could inform potential targets for anxiety treatment in autistic youth. The current study aims to examine the associations between CF and anxiety in this population. Given the complexity of CF as a multifaceted construct, we used a multi-method approach to measure CF in order to tease apart its relationship with anxiety symptoms. Specifically, we hypothesized that real-world flexibility difficulties (assessed by survey measures), and poorer task switching performance (assessed by neurocognitive lab measures), would be associated with higher anxiety.

**Methods:**

Participants included forty 8–17-year-old autistic children (11 female, 29 male), along with their parents. Anxiety symptoms were measured by parent- and self-report using the Anxiety Scale for Children with Autism Spectrum Disorder. Task switching performance was measured using the Trail Making, Verbal Fluency, and Color-Word Interference tests from the Delis–Kaplan Executive Function System, administered to children in a controlled lab setting. Flexibility challenges in real-world settings were assessed by parent- and self-report using the Flexibility Scale and Shift subscale from the Behavior Rating Inventory of Executive Functioning (BRIEF-2; BRIEF-SR). Autistic traits were measured by parent-report using the Social Responsiveness Scale (SRS-2).

**Results:**

Multiple linear regression analyses revealed that reduced real-world flexibility was a significant predictor of anxiety, according to both parent- and self-report, even after controlling for autistic traits. Poorer task switching performance, however, was not associated with increased anxiety.

**Discussion:**

Our findings are consistent with previous literature suggesting that real-world CF challenges are linked to anxiety in autistic youth. The current study also offers preliminary evidence for the distinction between real-world CF and performance-based CF regarding their relationship to anxiety among autistic youth. Findings may help inform targeted assessment and treatment approaches for anxiety in this vulnerable population.

## Introduction

Anxiety is among the most common and impairing problems experienced by autistic youth. Approximately 40% of youth with a diagnosis of autism spectrum disorder (ASD) meet criteria for an anxiety disorder (1, 2), and over 80% experience impairing anxiety symptoms (3). Autistic^1^ children are at significantly higher risk for developing anxiety disorders relative to non-autistic children (10). Moreover, those who engage in high autistic masking or remain undiagnosed may be susceptible to increased levels of anxiety, likely related to challenges with authenticity and self-esteem, as well as a lack of access to accommodations and support systems (11). Given the adverse impact of anxiety on adaptive functioning and quality of life (12–14), close monitoring of anxiety symptoms (e.g., pediatric mental health screenings, teacher observation, parent awareness) and early intervention efforts are critical for autistic youth. However, anxiety frequently gets overlooked and untreated in this population, in part, due to the unique and varying presentations of anxiety often observed in autistic youth (15, 16). For instance, unusual specific fears (e.g., supermarkets, happy birthday song), significant social distress (unrelated to fear of negative evaluation), and excessive worry about novelty have been documented as common ‘autism-specific’ symptoms of anxiety commonly seen in this population (17). Researchers have proposed that cognitive differences specific to autism, such as concrete thinking, may underlie the unique expressions of anxiety in autism (18). Thus, improved understanding of the cognitive mechanisms associated with anxiety in autistic youth may shed light on key risk factors and treatment targets, thereby improving screening and prevention of anxiety in autistic youth.

Cognitive flexibility (CF) has been posited as a potential cognitive substrate of anxiety vulnerability and anxiety symptom expression in autism (18–20). Given the concrete thinking styles and resistance to change commonly observed among autistic individuals (18–20), CF has long been a topic of interest in autism literature. Researchers have hypothesized that autistic differences in CF contribute to both unique strengths, such as a strong sense of justice (21), and mental health challenges, such as anxiety, in autistic individuals (18, 19).

CF has been defined as the ability to shift thoughts and behaviors according to situational demands (22). However, it has been operationalized in various ways across the literature. Most commonly, CF is measured using neurocognitive assessments or survey-based instruments. Neurocognitive assessments, typically administered in highly structured laboratory settings, are considered to be objective measures of an individual’s maximal ability. In contrast, survey measures, comprising of questions about flexibility challenges in real-world settings (e.g., switching between activities, adjusting to a change in plans, considering alternate points of view), aim to capture CF in everyday contexts. Current literature indicates that neurocognitive task performance and real-world surveys measure distinct constructs (23). Researchers increasingly conceptualize these as different facets of CF, highlighting the importance of multimethod approaches for comprehensive assessment of CF (24, 25).

The link between reduced flexibility (often referred to as *inflexibility* ^2^ in prior literature) and internalizing problems has been well established in the general population (27, 28). Individuals who experience affective symptoms, such as anxiety and depressed mood, often demonstrate inflexible thought and behavior patterns. At a cognitive level, it is common for these individuals to have repetitive and perseverative thoughts (i.e., rumination) and intrusive worries, with infrequent shifts in attention away from threatening or unpleasant thought content (29). Behaviorally, people with anxious/depressed phenotypes tend to display experiential avoidance, characterized by a persistent reluctance to engage in certain experiences (e.g., social isolation, staying in bed). These theoretical and observational accounts are supported by numerous empirical studies that have used task switching paradigms, showing that anxiety is specifically associated with the inhibition and switching components of CF (30).

While there is substantial literature establishing the link between poor performance-based CF and anxiety in general populations (e.g., 31–36), the potential role of CF in anxiety vulnerability specific to autistic youth warrants further investigation, given the observed overlap between reduced CF and common autistic traits (e.g., preference for sameness, black-and-white thinking, strong adherence to routines). Indeed, there are only a small number of existing studies, to our knowledge, that have examined this relationship in autistic samples. Hollocks et al. (37) demonstrated a negative association between task switching performance and parent-rated anxiety in a group of 90 autistic adolescents (aged 14–16 years), and Zimmerman et al. (38) reported a similar finding using self-ratings of anxiety in a sample of 42 autistic adults. Consistent with these cross-sectional findings, Hollocks et al. (39) went on to demonstrate this association across time points, in which poorer task switching performance at age 16 predicted greater parent-rated anxiety at age 23. In a more recent study, however, Conner et al. (40) found no significant relationship between task switching and self-reported anxiety in a sample of 57 autistic adolescents and adults (aged 16–25 years). Overall, there is limited research on how performance-based CF relates to anxiety in autistic people, especially among children. Given that executive function (EF) difficulties are more apparent during middle childhood and adolescence (41, 42), it may be particularly informative to investigate performance-based CF during these developmental periods in order to improve understanding of cognitive mechanisms of autism-specific anxiety. Moreover, task switching ability (i.e., performance-based CF) appears to be variable across autistic youth (43–47), in line with current conceptualizations of autistic cognition as a wide spectrum.

On the other hand, real-world flexibility difficulties may be more consistently prevalent among autistic individuals. Relative to non-autistic groups, significantly greater CF challenges have been found in autistic children, based on parent-report (42, 44), and in autistic adults, based on self-report (48). With the exception of CF, there appears to be wide variability across most domains of real-world EF (e.g., working memory, task completion, emotional control) among autistic youth (44). When comparing neurodevelopmental groups, autistic children have demonstrated greater CF difficulty than children with attention deficit/hyperactivity disorder (ADHD), Tourette’s syndrome, and traumatic brain injury (44, 48–50). These findings highlight a potential CF-specific vulnerability in autistic youth, which has prompted research interest in studying its role in the internalizing symptoms of this population. Two studies have documented a significant association between parent-rated CF difficulties and elevated anxiety levels in autistic children (48) and adults (51). In contrast, Gardiner and Iarocci (52) did not find a significant association between parent-rated CF and anxiety in a group of autistic children. The authors attributed these results to their use of a traditional anxiety measure that relies on the child’s verbal communication of their worries (e.g., says “I get nervous during a test”) and contains vague items (e.g., is fearful). Such symptoms are often difficult to identify in autistic youth due to difficulties in social communication and differences in anxiety expression (52, 53). Indeed, a number of studies have documented the prevalence of autism-specific symptoms of anxiety, which do not get captured by standard anxiety measures (15–17, 54, 55). This underscores the importance of using anxiety measures validated for autistic youth for more accurate assessment of anxiety in this population. Despite prior development of autism-specific anxiety measures (17, 54, 56), standard anxiety scales continue to be widely used in autistic youth. To our knowledge, there are no existing studies that have used an autism-specific anxiety scale to examine the relationship between CF and anxiety in an autistic sample.

Current literature provides preliminary evidence for a potential link between CF and anxiety in autistic youth, though the nature of this relationship remains inconclusive. Of the existing research examining this association, virtually no study has specifically assessed both performance-based and real-world aspects of CF within the same sample of autistic youth (39). In addition, only two preliminary studies, to our knowledge, have used autistic self-reports to measure anxiety in relation to CF (38, 40). Unfortunately, the majority of existing literature on anxiety in autistic youth has relied solely on parent- or teacher-report (2, 3), likely due to previous skepticism of the reliability of autistic youth self-reports (57), as well as documented challenges with communication and insight in autistic children (58). The lack of existing studies using autistic self-report measures is problematic, however, as caregiver-report alone is likely insufficient for capturing the internal experiences of autistic children (59, 60). Thus, further investigation involving multi-informant and multi-method approaches is necessary to gain a more comprehensive understanding of child anxiety and to characterize its link to CF in autistic individuals.

The current study extends the literature by investigating the relationship between CF and anxiety in autistic youth using both performance-based tasks and real-world survey measures of CF. Importantly, this study leverages the Anxiety Scale for Children with Autism Spectrum Disorder (ASC-ASD; 54), a tool designed to capture the distinct anxiety profiles of autistic youth from their own perspectives and their parents’ perspectives. Drawing on previous preliminary findings and theoretical accounts (37, 48, 51, 61), we hypothesized that greater real-world flexibility challenges and poorer task switching performance would be associated with higher levels of anxiety. By testing these associations, the present study aims to advance our conceptual understanding of the link between CF and anxiety specific to autistic youth.

## Materials and methods

### Participants

Participants (*n* = 40) came from a subject pool of autistic and non-autistic children participating in a larger research study on the neural mechanisms of CF, which took place at a university in northeastern United States. These participants were recruited from various community centers, schools, and clinics through word of mouth, flyers, emails, and general information sessions. Participants in the current study were administered questionnaires and neuropsychological measures by trained graduate students. Parents of participants also completed questionnaires. Study data were collected and managed using REDCap electronic data capture tools (62, 63).

Participants were selected for the present study based on their age (8–17 years), verbal ability (i.e., having a strong grasp of expressive language), reading ability (i.e., could accurately identify letters, read and understand simple words), and autism spectrum diagnostic criteria. The Autism Diagnostic Observation Schedule (ADOS-2; 64) was administered by research-reliable examiners to determine diagnostic eligibility; only participants with an ADOS-2 classification of Autism or Autism Spectrum were included. A total of 40 participants met eligibility criteria and were included in the present study.

### Measures

#### Demographics

Parents completed a background questionnaire in which they provided their children’s demographic information, including age, sex, gender, and race. They also answered questions about whether their child had previously received a psychiatric diagnosis.

#### Anxiety symptoms

The Anxiety Scale for Children with Autism Spectrum Disorder (ASC-ASD; 54) is a survey measure designed to assess anxiety in autistic youth. The validity of the parent- and self-report forms of this measure has previously been demonstrated in multiple samples of autistic youth (54, 65–67). It comprises 24 items and assesses both typical (15 items) and atypical (9 items) symptoms of anxiety. The child or informant rates the frequency of each symptom on a four-point Likert-scale from 0 (*Never*) to 3 (*Always*). The ASC-ASD includes four subscales: Uncertainty; Performance Anxiety; Separation Anxiety; and Anxious Arousal. The total score is a sum of all 24 items and can range between 0 and 72. Total raw scores of 20 or higher are indicative of clinically elevated levels of anxiety (54, 68). Total raw scores were examined in descriptive analyses to determine the number of participants with clinically elevated scores. In addition, per item mean scores^3^ were calculated based on total and subscale raw scores, allowing for comparison of subscales since each subscale has a different number of items. Total per item mean scores from both the parent- and self-report measures were the outcome variables in primary analyses.

#### Autistic traits

The Social Responsiveness Scale School-Age Form (SRS-2; 69) measures social differences characteristic of autism and is commonly used as an autism screener. The SRS-2 consists of 65 items which are rated on a four-point Likert-type scale, ranging from 1 (*Not True*) to 4 (*Almost Always True*). This measure has shown strong sensitivity, as well as specificity in differentiating between groups within the autism spectrum and between autistic and other neurodivergent groups (70). SRS-2 total raw scores were converted to *T*-scores using norms based on age and gender. Higher *T*-scores indicate stronger and more frequently occurring autistic characteristics.

#### Real-world flexibility

The Behavior Rating Inventory of Executive Functioning (BRIEF; 71) is used to measure behaviors in unstructured, natural settings as they correspond to higher-level cognitive abilities, or EF. In particular, the shift subscale provides an ecologically valid index of CF, as items on this scale assess flexible problem solving, change tolerance, attention switching, transition making, and focus shifting in daily life. The shift subscale consists of eight items, in which the child or caregiver rates frequency of behaviors on a 3-point Likert-type scale (i.e., *Never, Sometimes, Often*) during the last six months. The caregiver-report version (BRIEF-2) has been validated for youth (ages 5-18), and the self-report version (BRIEF-SR) has been validated for ages 11 to 18 (71). Shift subscale *T*-scores from the BRIEF-2 and BRIEF-SR were used in data analyses.

The Flexibility Scale (FS; 72) is a parent/caregiver report measure used to assess CF in real world settings. The FS consists of 27 items, in which the caregiver rates behaviors on a 4-point Likert-type scale (i.e., *No*, *Somewhat*, *Very much*, *Always*) during the last two weeks. It has a five-factor structure: routines/rituals, transitions/change, special interests, social flexibility, and generativity. The FS has shown promise as a comprehensive measure of real-world flexibility, as it has demonstrated good psychometric properties and similar construct validity as the BRIEF Shift subscale (72). FS total raw scores were used in analyses. Higher scores indicate lower CF.

#### Performance-based task switching

Neurocognitive measures are used to objectively assess behavioral responses in a highly structured setting and are generally indicative of maximal cognitive ability (73). The Delis–Kaplan Executive Function System (D-KEFS; 74) is a comprehensive set of standardized performance measures that assess EFs, with normative data from a large U.S. sample (ages 8-89). The D-KEFS tests have demonstrated reliability and validity as indices of EF across numerous studies (74–77). Each test consists of multiple conditions with the purpose of isolating specific EFs from other cognitive processes (e.g., attention, language, concept formation) involved in task performance. D-KEFS tests, such as those described below, are often used to assess performance-based CF, as they each include a *switching* condition, which involves switching between tasks under timed conditions. Contrast scaled scores were calculated to measure performance differences between switching conditions and baseline conditions in order to isolate CF from the other cognitive abilities involved in each test.

Verbal Fluency. The D-KEFS Verbal Fluency (VF) test measures the ability to generate certain words as quickly as possible. It comprises three conditions: *letter fluency, category fluency*, and *category switching*. The latter two conditions, which were used in the current study, are semantic retrieval tasks, in which the participant must generate words from a specific semantic category (e.g., animals). In the switching condition, the participant must alternate between two different semantic categories (e.g., fruits and furniture). Scaled scores were calculated for total correct responses and total switching accuracy. Category switching vs. category fluency contrast scores were also calculated and examined. Higher contrast scores indicate better switching performance.

Trail Making Test. The D-KEFS Trail Making Test (TMT) is a timed visual-motor sequencing task. It comprises five conditions: *visual scanning, number sequencing, letter sequencing, number-letter switching*, and *motor* sp*eed*. The switching condition requires participants to alternate between connecting numbers and letters in ascending order (i.e., 1-A-2-B-3-C). The scaled score for the time to complete the switching condition was calculated. Contrast scaled scores were also used to measure switching performance while accounting for motor speed, visual scanning, and sequencing abilities.

Color-Word Interference. The D-KEFS Color-Word Interference (CWI) test is based on the Stroop task (1935). It consists of four conditions: *color naming* (naming color patches), *word reading* (reading words that denote colors and printed in black ink), *inhibition* (inhibiting the more automatic response to read words denoting colors and instead naming the incongruous ink colors that the words are printed in), and *switching*. The switching condition requires participants to switch between naming the incongruous ink colors and reading the words, depending on whether the word is in a box, as quickly as possible (74). Relative to the previously described switching tasks, this task is considered more difficult, as it involves “perceptual switching.” In other words, the participant must pay attention to stimulus features in order to switch appropriately (i.e., if word is boxed) and respond appropriately (with ink color or word), rather than simply alternating between two tasks (e.g., naming fruit and furniture in Verbal Fluency task). The scaled score for the time to complete the switching condition was calculated. Switching contrast scores were also calculated to examine switching performance while accounting for naming, reading, and inhibition abilities.

### Data analytic plan

Data preparation and analyses were conducted using IBM SPSS Statistics (Version 29.0.2.0). The study data were checked for missingness and outliers. Item-level missing data were addressed by following the guidelines specified by each scale’s manual. No participant missed more than three items on any given scale. Descriptive statistics were computed for all study variables. A paired samples *t*-test was conducted to test the significance of the discrepancy in total anxiety scores between informants (parent- vs. self-report). Preliminary bivariate relationships between all study variables were assessed using Pearson’s correlation.

#### Regression analyses

Hierarchical linear regression analyses were conducted to fulfill the study aim of assessing how real-world CF and performance-based CF relate to anxiety. Parent-reported and self-reported total anxiety scores were the outcome variables in regression analyses. Preliminary correlations were used to guide the selection of predictor variables to include in each regression model. Real-world CF ratings and performance-based CF scores that had significant bivariate relationships with anxiety outcomes (*p* <.05) were carried forward in the regression analyses. In addition, age, sex, IQ, and autistic trait score were examined in preliminary analyses to determine which participant characteristics to include in regression models as covariates, based on significant bivariate correlations with outcome anxiety variables (*p* <.05). Listwise deletion was used for missing data. Thus, five participants who were lost to follow-up and did not complete CF measures were excluded from regression analyses. All independent variables were mean centered for regression analyses. Assumptions for all linear regression analyses were checked.

## Results

### Descriptives

Participant characteristics of the study sample (*N*=40) are provided in **Table 1**. The sample was predominantly male (72.5%), with a mean age of 12.1 (*SD*=3.0) at the start of the study. Race and ethnicity varied, with 47.5% White and 52.5% non-Hispanic/Latinx. Mean IQ was 106.6 (*SD=*16.8), as measured by the Kaufman Brief Intelligence Test (KBIT-2; 78). Half of the participants (*N*=20) had a diagnosis of ADHD, based on retrospective parent-report. Descriptive statistics of all study variables are presented in **Table 2**.

**Table 1:** Participant Characteristics (N = 40).

Variable	*N*	%	*M* (*SD*)
Age ^a^			12.1 (3.0)
IQ ^b^			106.6 (16.8)
Biological Sex			
Female	11	27.5	
Male	29	72.5	
Gender Identity			
Girl/Woman	13	32.5	
Boy/Man	26	65.0	
Non-binary	1	2.5	
Race			
Asian	7	17.5	
Black	5	12.5	
Middle Eastern/North African (MENA)	3	7.5	
White	19	47.5	
Multiracial ^c^	2	5.0	
Not disclosed	4	10.0	
Ethnicity			
Hispanic/Latinx	6	15.0	
Non-Hispanic/Latinx	21	52.5	
Not disclosed	13	32.5	
Caregiver Relationship			
Mother	37	92.5	
Father	3	7.5	
Co-occurring Diagnoses ^d^			
ADHD	20	50	
Learning Disorder	5	12.5	
Language Disorder	1	2.5	
Intellectual Disability	3	7.5	
Depression	4	10	
Anxiety	8	20	
Adjustment Disorder	1	2.5	
PTSD	2	5	
ODD	3	7.5	

a Age at the start of study.

b Kaufman Brief Intelligence Test (KBIT-2) IQ composite standard score.

c These participants were identified by their parents as the following: 1) Asian and White, and 2) Black, American Indian/Native Alaskan, and White.

d Psychiatric diagnoses based on retrospective caregiver-report.

**Table 2:** Descriptive statistics of study variables.

Variable	*N*	*M*	*SD*	Range
BRIEF-2: Shift (*T*-score)	35	67.54	13.67	43-91
BRIEF-SR: Shift (*T*-score)	25	67.16	10.26	45-91
Flexibility Scale-Revised: Total (raw score)	35	30.97	11.19	9-52
SRS-2: Total (*T*-score)	40	66.65	12.37	40-90
ASC-ASD-P (per item mean scores)				
Performance Anxiety	40	0.77	0.66	0-2.40
Anxious Arousal	40	0.21	0.27	0-1.33
Separation Anxiety	40	0.49	0.52	0-2.00
Uncertainty	40	0.67	0.52	0-1.88
Total	40	0.53	0.37	0-1.42
ASC-ASD-C (per item mean scores)				
Performance Anxiety	39	1.24	0.77	0-3.00
Anxious Arousal	39	0.75	0.68	0-2.33
Separation Anxiety	39	0.92	0.60	0-2.20
Uncertainty	39	1.10	0.66	0-2.38
Total	39	1.00	0.55	0-2.13
D-KEFS Trail Making Test (scaled scores)				
Switching	35	8.03	3.88	1-14
Switching vs Visual Scanning	35	9.46	3.80	1-19
Switching vs Combined Number/Letter	35	9.23	3.41	1-17
Switching vs Motor Speed	35	9.51	3.73	1-19
D-KEFS Verbal Fluency (scaled scores)				
Category Switching Total Correct Responses	36	8.69	2.51	4-13
Total Switching Accuracy	36	9.00	2.70	1-14
Category Switching vs Category Fluency	36	9.64	3.04	3-16
D-KEFS Color-Word Interference (scaled scores)				
Inhibition/Switching	35	8.40	4.17	1-35
Inhibition/Switching vs Combined Naming/Reading	35	9.71	3.04	3-35
Inhibition/Switching vs Inhibition	34	8.76	3.25	1-34

#### Anxiety symptoms

On the ASC-ASD, parent-reported total raw scores ranged from the minimum to 34, with a mean of 12.75 (*SD*=8.81), while self-reported total raw scores ranged from the minimum to 51, with a mean of 24.10 (*SD*=13.15). Seven participants (17.5%) had parent-reported total raw scores of 20 or higher, a cut-off that has been shown to be indicative of significant levels of anxiety (54, 68). In contrast, more than half of the participants (22; 56.4%) had self-reported total raw scores above this cut-off. There was a statistically significant difference between parent- and self-ratings of anxiety [*t*(38) = - 5.54, *p* <.001].

In addition, per item mean scores were calculated in order to compare the mean subscale scores of the sample (**Table 2**). For both parent- and self-report, scores in the Performance Anxiety domain were the highest, followed by Uncertainty, then Separation Anxiety, and lowest for Anxious Arousal.

#### Cognitive flexibility

With regards to real-world flexibility, parent-ratings on the BRIEF-2 Shift subscale indicated clinically elevated CF difficulties (*T*-score of 65 or higher; 71) in about 68.6% of participants. Self-ratings on the BRIEF-SR Shift subscale indicated 64% of participants with clinically elevated CF difficulties. On D-KEFS measures of CF, contrast scaled scores of 7 or lower reflect disproportionately worse performance on the switching condition (74), indicating possible performance-based CF challenges. On the TMT, five participants out of 35 demonstrated worse switching performance relative to number and letter sequencing performance. On VF, nine participants out of 36 demonstrated worse category switching performance relative to category fluency. On CWI, seven participants out of 34 demonstrated worse switching performance relative to performance on baseline naming and reading conditions, and eight out of 34 participants demonstrated worse switching performance relative to the inhibition condition.

### Bivariate correlations

**Figure 1:**
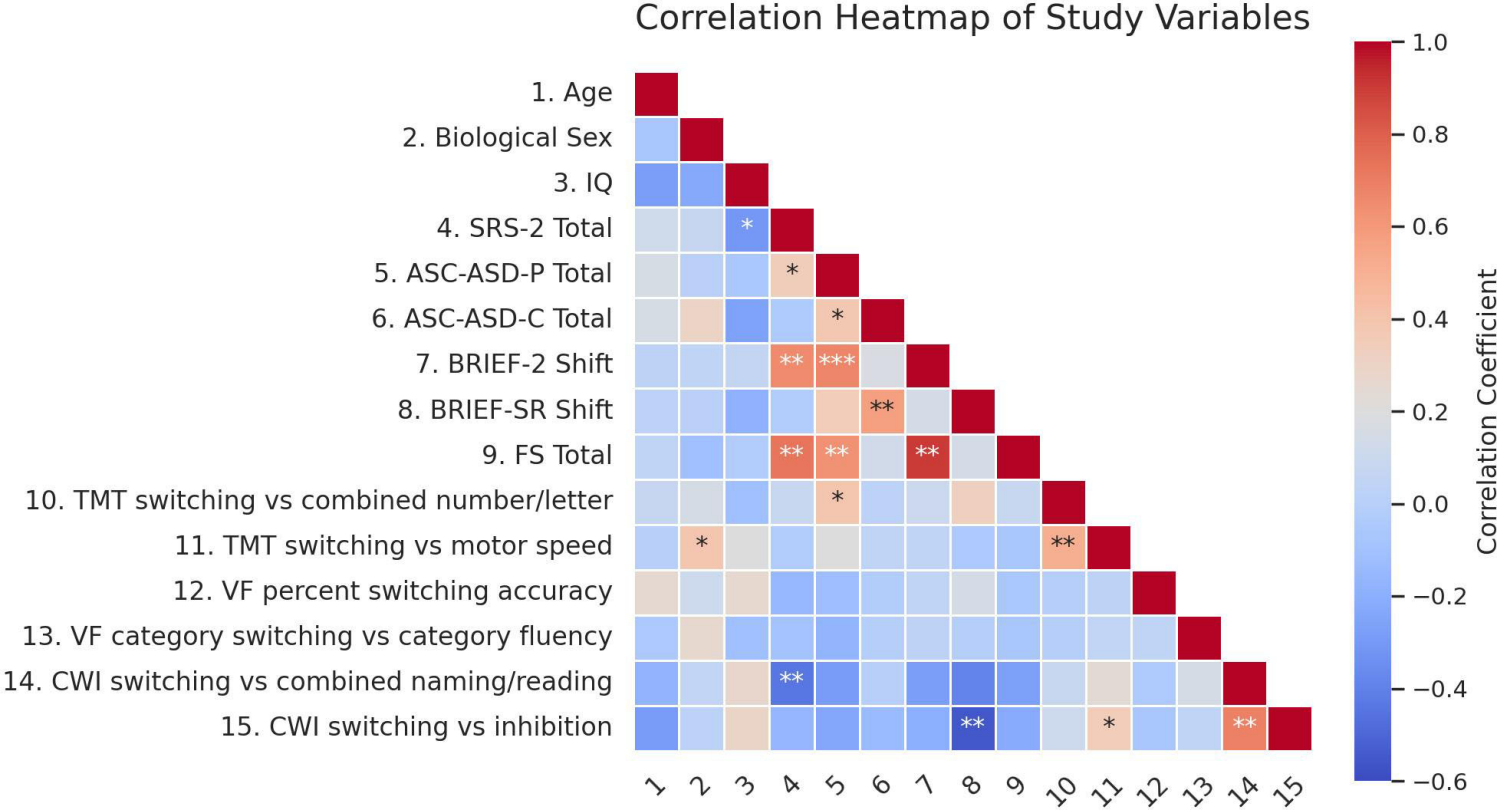
Red hues indicate negative correlations, blue indicate positive correlations, and light shades indicate weak correlations. Asterisks indicate significance levels (*p<0.05, **p<.01, ***p<.001).

Prior to conducting regression analyses predicting total anxiety, bivariate correlations were examined to inform the selection of independent variables to include in hierarchical regression analyses. A correlation heatmap is displayed in **Figure 1**.

Autistic trait score was moderately correlated with parent-reported total anxiety (*r*=.34, *p*=.034). Other participant characteristics (i.e., sex, age, IQ) did not significantly correlate with anxiety outcomes. Thus, autistic trait score was included in regression analyses as a covariate, in order to examine relationships between CF and anxiety outcomes, controlling for autistic traits.

Across the three D-KEFS tests, only TMT switching performance (Switching vs. Combined Number and Letter contrast scaled score) was significantly correlated with parent-reported total anxiety. However, the bivariate association was opposite to the study hypothesis, such that better switching performance was associated with higher anxiety (*r*=.39, *p*=.021). None of the task switching scores across the three D-KEFS tests were significantly associated with self-reported total anxiety.

The BRIEF-2 Shift subscale score and FS total score both demonstrated strong correlations with parent-reported total anxiety (*r*=.67, *p*<.001; *r*=.63, *p*<.001). Similarly, the BRIEF-SR shift subscale score demonstrated a strong bivariate correlation with self-reported total anxiety (*r*=.57, *p*=.003).

### Regression findings

#### Parent-reported anxiety

A hierarchical regression analysis was conducted to predict parent-reported total anxiety. Model 1 included autistic trait score. In Model 2, parent-reported real-world flexibility (FS total score) and task switching performance (TMT Switching vs. Combined Number/Letter contrast score) were entered. Model 1 was statistically significant [*F*(1, 32) = 6.83, *p* = .014], with higher autistic trait score predicting higher anxiety. When CF variables were added in Model 2, the model remained statistically significant [*F*(2, 30) = 11.00, *p* <.001] and accounted for 52.4% of the variance in parent-reported total anxiety. FS score was a significant unique predictor, such that higher real-world flexibility predicted lower anxiety (*B* = 0.02, *p* <.001), as expected. TMT switching performance, however, had an opposite relationship with anxiety, such that better switching performance was significantly associated with higher anxiety (*B* = 0.04, *p* = .010; **Table 3**). A *post hoc* power analysis using G*Power indicated that, with an observed *R*² of .524 (f² ≈ 1.10), α = .05, *N* = 35, and three predictors, the achieved statistical power to detect the observed effect was greater than 0.99, indicating excellent sensitivity to detect this effect size.

**Table 3:** Results of the hierarchical linear regression model predicting parent-reported total anxiety (N=35).

Effect	Model 1		Model 2	
	*B*	*SE*	*B*	*SE*
Constant	0.57***	0.06	0.54***	0.05
SRS-2^a^	0.01*	0.01	-0.004	.01
FS^b^			0.02***	0.01
TMT^c^			0.04*	0.01
R^2^	0.176*		0.524***	

*p<0.05, ***p<.001.

a Social Responsiveness Scale (SRS-2) Total score.

b Flexibility Scale (FS) Total score.

c Trail Making Test (TMT) Switching vs. Combined Number/Letter contrast score.

A second regression analysis predicting parent-reported total anxiety was conducted, in which BRIEF-2 Shift score was entered as a predictor. Similar to the results of the first regression analysis, BRIEF-2 Shift score was a significant unique predictor of parent-rated anxiety (*B* = 0.02, *p* < .001; **Table 4**).

**Table 4:** Results of the hierarchical linear regression model predicting parent-reported total anxiety (N=35).

Effect	Model 1		Model 2	
	*B*	*SE*	*B*	*SE*
Constant	0.57***	0.06	0.55***	0.05
SRS-2^a^	0.01*	0.01	-0.001	.01
BRIEF-2 shift ^b^			0.02***	0.004
TMT^c^			0.04*	0.01
R^2^	0.176*		0.553***	

*p<0.05, ***p<.001.

a Social Responsiveness Scale (SRS-2) Total score.

b Behavior Rating Inventory of Executive Functioning (BRIEF-2) Shift score.

c Trail Making Test (TMT) Switching vs. Combined Number/Letter contrast score.

#### Self-reported anxiety

A hierarchical regression analysis was conducted predicting self-reported total anxiety. It should be noted that a subset of the study sample (N=25) was included in this analysis since not all participants met age criteria (i.e., ages 11 and older) to complete the BRIEF-SR. Autistic trait score was entered in Model 1. Self-reported real-world flexibility (BRIEF-SR Shift) and task switching performance (CWI Switching vs. Inhibition contrast score) were added in Model 2. As shown in Model 1, autistic trait score was not significantly associated with self-reported anxiety. Model 2 was statistically significant [*F*(1, 22) = 10.35, *p* = .004] and accounted for 32.7% of the variance in anxiety. Higher real-world flexibility was significantly associated with lower self-reported total anxiety (*B* = .03, *p* = .004), independent of autistic traits (**Table 5**).

**Table 5:** Results of the hierarchical linear regression model predicting self-reported total anxiety (N=25).

Effect	Model 1		Model 2	
	*B*	*SE*	*B*	*SE*
Constant	1.08***	.11	1.08***	0.09
SRS-2^a^	-0.004	0.01	-0.003	0.01
BRIEF-SR shift ^b^			0.03**	0.01
R^2^	0.011		0.327**	

**p<.01, ***p<.001.

a Social Responsiveness Scale (SRS-2) Total score.

b Behavior Rating Inventory of Executive Functioning (BRIEF-SR) Shift score.

## Discussion

This study extends the current literature by its use of a multi-method approach to investigate the relationship between CF and anxiety in autistic youth. To our knowledge, this is one of the first studies to examine this link using both survey measures and task performance measures of CF in the same sample of autistic youth, allowing for a more comprehensive investigation of the link between CF and anxiety.

### Real-world flexibility and anxiety

Findings supported the hypothesized positive association between reduced real-world flexibility and greater anxiety in autistic youth, based on both parent- and self-reports. Specifically, parent-reported flexibility difficulties predicted greater parent-reported overall anxiety, independent of autistic traits. These results are consistent with prior autism studies that have found parent-reported real-world flexibility difficulties to be significantly associated with parent-reported anxiety (48, 51, 61). Youth self-reports yielded similar findings. Self-rated flexibility difficulties predicted greater self-reported anxiety, when controlling for autistic traits. These preliminary findings support recent theoretical accounts and emerging evidence proposing low CF as a potential mechanism of anxiety vulnerability in autism. Specifically, difficulty deviating from internal expectations or plans may relate to increased uncertainty and subsequent anxiety (61, 79).

Our study adds to this literature, as it is the first to use autistic youth self-report of flexibility challenges to investigate the relationship between CF and anxiety. The positive associations found in the current study between reduced self-reported flexibility and increased self-reported anxiety yielded similar effect sizes as those found using parent-report measures. Importantly, we observed a discrepancy in the magnitude of anxiety ratings between parent- and self-report in our sample, as autistic youth rated their own anxiety symptoms as significantly greater than parents’ ratings of their symptoms. Taken together, these findings suggest the utility of asking autistic youth and their caregivers about flexibility challenges in daily life as a means of assessing anxiety vulnerability in autistic youth, regardless of informant agreement on anxiety levels.

It should be noted that the parent-child discrepancy observed in this study is in line with the broader anxiety literature on the general pediatric population (e.g., 80–83). However, it is inconsistent with findings from previous studies using the ASC-ASD in clinical samples of autistic youth (54, 68). We attributed the informant discrepancy to our use of a community-based sample, as it would naturally comprise of more subclinically anxious youth than those with co-occurring anxiety diagnoses, compared to clinic-referred youth. That is, parents may be less likely to recognize internalizing symptoms that may not be severe enough to warrant a clinical diagnosis. This is supported by a recent study which documented child anxiety ratings exceeding parent ratings in a group of autistic youth without anxiety disorders but not in a group of autistic youth with anxiety disorders (84). In line with this, Ooi et al. (60) found that parent-child agreement of anxiety was associated with symptoms involving clearly observable behaviors. Thus, our preliminary findings support the use of self-report to gain a more comprehensive understanding of anxiety in autistic youth, especially outside of clinical settings where child anxiety tends to be less apparent and more representative of general autistic child populations (60, 84). This study also lends credence to mounting criticism of a common assumption among clinicians and researchers that autistic youth lack the cognitive capacity and insight to self-reflect on their symptoms (3, 85).

### Performance-based task switching and anxiety

Our findings did not support the hypothesis that poorer performance-based CF predicts greater anxiety in autistic youth. Switching performance scores across three neurocognitive tests (i.e., TMT, VF, CWI) were used as measures of performance-based CF. None of the task switching measures were significantly associated with self-reported anxiety. This finding is consistent with a recent study that found no association between switching performance on the TMT or CWI test and self-reported anxiety in a sample of 57 autistic adolescents and young adults (40). Regarding parent-reported anxiety, number-letter switching (TMT) performance was a significant correlate, while VF and CWI switching measures were not. Unexpectedly, however, better TMT switching performance was associated with greater parent-reported anxiety. In contrast, prior studies have demonstrated weak-to-moderate associations between poorer TMT switching and greater parent-reported anxiety in autistic youth (37, 39). We posit that this inconsistency may be attributed to differences in sampling. Our findings are based on a community sample while these prior studies used clinical samples with anxiety diagnoses, naturally characterized by higher levels of anxiety than those of a community sample. It is possible that an association between poor task switching performance and anxiety can only be captured when anxiety symptoms are at clinically significant levels.

The unexpected positive association between TMT switching performance and parent-reported anxiety may be understood within the framework of attentional control theory (ACT; 86). According to ACT, individuals with elevated anxiety allocate attentional resources away from goal-driven behavior (i.e., task performance) toward threat-related stimuli. In addition, ACT posits that task accuracy can be buffered by additional mental effort; however, this compensatory strategy comes at the cost of task efficiency. Several studies have empirically supported the moderating role of mental effort on the relationship between anxiety and task performance in non-autistic populations (87, 88). There is also evidence to suggest that high state anxiety (i.e., situational stress) adversely impacts switching performance only on high-complexity tasks, not on low-complexity tasks (89). Based on this theory and research, we speculate that the more anxious participants in our overall subclinical community sample demonstrated a similar phenomenon. That is, they may have employed extra effort on all switching tasks, thereby buffering the impact of CF difficulties on task switching performance. Additionally, it is important to note that the TMT is lowest in complexity relative to the other task switching paradigms used in the current study, as it does not require perceptual switching like in CWI or semantic retrieval like in VF. Given that TMT is a low-complexity switching task, it is possible that the additional effort deployed by the high anxiety participants did not lead to a meaningful cost in performance efficiency but rather resulted in overall enhanced task performance on TMT switching. Therefore, our unexpected finding that TMT switching performance was associated with greater anxiety may be attributed to the low complexity of the TMT switching task and the potential buffering role of mental effort on task performance. Future studies assessing performance-based CF should include a measure of mental effort, such as the Rating Scale of Mental Effort (90), as well as multiple task switching paradigms of varying levels of complexity, in order to test these hypothesized effects of mental effort and task complexity on performance-based CF.

### Clinical implications

In the current study, survey ratings of real-world flexibility challenges were consistently associated with increased anxiety, while task switching performance was not. These findings are consistent with prior literature indicating a lack of convergence between these two types of CF measures in autistic (91) and general populations (23, 92–94). Leung and Zakzanis (91) conducted a meta-analysis of 72 studies that measured CF in autistic populations and did not find evidence for an association between BRIEF-2 Shift scores and task switching performance. In spite of the common usage of task switching measures by clinicians and researchers to assess the day-to-day challenges with flexibility of autistic youth (19), there is growing skepticism regarding the ecological validity of these performance-based CF measures (19, 95). Based on our findings and the existing literature, we urge researchers and clinicians to use a multi-method approach to assessing CF that includes informant- and self-reports.

Additionally, the lack of association between task switching and anxiety in the current study suggests that this performance-based facet of CF may be a less meaningful treatment target among autistic youth with anxiety. Thus, interventions designed to improve neurocognitive abilities, such as cognitive remediation therapies (CRT), may not be most beneficial for improving mental health outcomes of autistic youth. There is emerging evidence indicating the efficacy of CRT in improving performance-based CF in autistic people (96–98). Based on our preliminary findings, however, we suggest that improvement in this form of CF may not have a therapeutic impact on anxiety in autistic youth. Furthermore, Simons et al. (99) found that cognitive trainings can help to improve performance on the specific tasks being trained but that these benefits do not generalize to daily life skills. Additional research investigating a possible causal link between task switching ability and mental health outcomes is necessary to determine the clinical utility of CRT for treating autistic youth with anxiety or other mental health concerns.

Our study offers support for the potential role of real-world flexibility difficulties in the development and maintenance of anxiety in autistic youth, which has previously been proposed by researchers (61, 79). Based on our findings on real-world CF, we suggest the potential mental health benefits of EF interventions that target flexibility challenges in daily life. The *Unstuck and On Target* (UOT) intervention is one such example, as it was developed for teachers, caregivers, and service providers to support autistic youth flexibly manage day-to-day challenges, like handling schedule changes, compromising with peers, and adjusting a plan when needed (100, 101). A recent pilot trial examining the feasibility and impact of UOT adapted for community mental health settings provided preliminary support for the intervention’s potential to improve mental health symptoms of autistic youth (102).

In addition, the literature has established the importance of environmental modifications for supporting autistic youth with real-world flexibility challenges. Empirical evidence from inclusive classrooms have demonstrated the significant impact that predictable routines (e.g., using visual schedules), auditory cues (e.g., using songs to facilitate transitions), physical engagement (e.g., giving opportunities for movement), and other environmental modifications can have in promoting autistic students’ flexibility and self-regulation skills (103). Importantly, the everyday flexibility challenges and associated stress and anxiety faced by many autistic people often stem from a mismatch between the neuronormative expectations of the social environment and the innate neurological functioning of the individual. Thus, there is a need for research that seeks to better understand the socioenvironmental barriers to autistic well-being (e.g., schedules, curricula, environmental design, stigma). Furthermore, shifting the focus away from individual psychopathology to the broader environmental context can promote autistic mental health by reducing social stigma and alleviating the stress associated with conforming to social norms. As indicated by recent qualitative findings, conforming to fit into neurotypical environments (i.e., “autistic masking”) may have detrimental effects on the self-esteem and sense of identity for autistic adolescents and adults (104–106). Therefore, when developing and implementing CF interventions, it is important for researchers and service providers to be mindful not to inadvertently focus on traits or behaviors that may simply reflect individual differences. In contrast, prioritizing efforts to build inclusive and affirming environments may be most effective in facilitating skill development and ultimately promoting the long-term mental health and well-being of autistic individuals.

### Limitations and future directions

Study findings should be interpreted with consideration of several limitations. Our study excluded autistic youth with severe language difficulties in verbal expression, due to the verbal requirements of the measures we used. As a result, our sample is not representative of the full spectrum of cognitive and language functioning in autism, thus limiting generalizability of the findings to all autistic populations. The lack of gender and racial diversity in our sample also limits generalizability.

In addition, the cross-sectional study design limited our ability to assess causality between CF challenges and anxiety. The only existing longitudinal study to examine this relationship in an autistic sample found that performance-based CF in adolescence was linked to anxiety and other mental health difficulties in young adulthood (39). Future studies should investigate long-term anxiety outcomes associated with both real-world and performance-based CF across developmental stages. A developmental trajectory approach can help advance our mechanistic understanding of how CF may contribute to the emergence and persistence of anxiety in autistic youth. We also recommend the inclusion of a comparison group in future research. A non-autistic comparison group would allow for better understanding of the unique effects of autistic traits on CF and its associations with anxiety.

The methods of data collection used in the current study consisted of both strengths and limitations. The use of a multi-informant approach inclusive of autistic self-report was a strength of the current study, as research has demonstrated the importance of self-report in comprehensive and accurate understanding of the internal experiences of autistic youth (59, 60, 107). However, validity of self-report findings may have been influenced by potential limitations in children’s self-awareness and possible developmental delays (58). In addition, parental ratings of anxiety may have been biased by parenting stress (60). As feasible, future studies should opt for semi-structured interviews, which are more reliable and valid than survey measures in assessing psychopathology (108, 109) and less susceptible to response biases. In addition, the use of more objective measures of real-world CF, such as social or affective flexibility tasks or natural observation, may offer a promising approach to less biased measurement of CF while still maintaining ecological validity.

It is also possible that common method bias may have impacted validity of our findings (i.e., real-world flexibility associated with anxiety due to common source of measurement rather than a true association between constructs). However, given the time delay between administering anxiety surveys and CF measures for most participants, we suspect that common method bias had minimal impact. A mixed-methods approach is another important direction for future research, as it would allow for better understanding of the lived experiences of autistic youth and capture their unique perspectives regarding their day-to-day challenges with flexibility. In-depth interviews, focus groups, and naturalistic observations have the potential to provide valuable insights and add nuance to quantitative findings, which in turn, could help inform clinical applications of this research.

Measurement of performance-based CF comes with its own set of challenges. Due to the ‘task impurity problem,’ it is impossible to completely isolate CF from other cognitive functions (110); thus, the construct validity of any task switching paradigm is inherently limited. The current study also did not control for state anxiety which may have impacted task switching performance (19, 111). Future studies with larger samples can randomize the order of tasks in order to account for potentially elevated state anxiety at the start of neurocognitive assessments. State anxiety may also be assessed and controlled for using physiological measures of arousal such as skin conductance response or salivary cortisol. In addition, we did not account for perceived self-efficacy which likely has a direct impact on task switching performance, regardless of underlying cognitive skill (112).

Further investigation is needed for a more comprehensive understanding of the mechanisms contributing to anxiety in autistic youth, as shown by the residual variance in our regression analyses. While executive functioning and social communication difficulties accounted for a substantial proportion of variance in anxiety, particularly in parent reports, there remains meaningful unexplained variance. For parent-reported anxiety, caregiver-related factors, such as parenting stress, caregiver anxiety, and family accommodation behaviors, may influence both the child’s emotional functioning and the parent’s interpretation of anxiety symptoms. For self-reported anxiety, other individual factors not measured here, such as emotion regulation capacity, sensory processing differences, and internal coping strategies, may help explain the remaining variance. Future studies that integrate both child- and parent-level variables may offer a more complete picture of the underlying processes involved in anxiety vulnerability of autistic youth.

## Conclusions

The current study aimed to deepen our understanding of the relationship between CF and anxiety in autistic youth. Despite its limitations, this study extends the current literature and highlights areas for future research needed to advance understanding of cognitive mechanisms of anxiety in autism. Results from this study can help to inform screening, prevention, and intervention approaches for anxiety in autistic youth. Our findings suggest that everyday challenges with flexible thinking may play a role in anxiety vulnerability and thus may be a beneficial treatment target for autistic youth at risk of developing anxiety. However, intervention efforts should also focus on addressing the socioenvironmental barriers contributing to the CF-related challenges in the lives of autistic individuals. Unlike real-world CF, performance-based CF may not be closely linked to anxiety in autistic youth, though additional empirical evidence is needed to support this. Future work involving longitudinal research will be important for elucidating any causal role and long-term impact of CF difficulties on the development and maintenance of symptoms of anxiety in autistic youth.

## Data Availability

The raw data supporting the conclusions of this article will be made available by the authors upon reasonable request to the corresponding author.

## Footnotes

1 While there is debate over whether person-first (‘person with autism’) or identity-first language (‘autistic person’) is most appropriate to describe individuals in the autism community, many survey studies have shown that autistic people most commonly prefer identity-first language (4–7), as it is generally perceived as less stigmatizing and more affirming of autism (8). In line with current consensus on inclusive language practices (9), we will use identity-first language in this paper henceforth except when referring specifically to the diagnosis.

2 We opted not to use the term ‘inflexibility’ in this paper, as it may reflect a deficit view of autism. In efforts to promote acceptance of autistic people, autism scholars and self-advocates have recommended shifting away from traditional medical language to describe autism in favor of using neurodiversity-affirming language (26).

3 ASC-ASD per item mean scores excluded any missing items.
